# “Of course, you get depression in this situation”: Explanatory Models (EMs) among Afghan refugees in camps in Northern Greece

**DOI:** 10.1186/s12888-023-04613-2

**Published:** 2023-02-27

**Authors:** Michalis Lavdas, Eugene Guribye, Gro Mjeldheim Sandal

**Affiliations:** 1 Department of Psychosocial Science, Faculty of Psychology, University of Bergen, Bergen, Norway; 2 NORCE Norwegian Research Centre, Kristiansand, Norway

**Keywords:** Explanatory Models, Afghan refugees, Refugee camps, Mental health, Depression, Trauma, Coping, Gender differences, Post Migration Living Difficulties

## Abstract

**Background:**

Afghan refugees and asylum seekers constitute one of the largest groups that live in camp settings in Greece. While they experience psychological distress, they are facing significant barriers in accessing appropriate mental health care. Explanatory Models (EMs) provide a context-sensitive framework for understanding the differences between health professionals, refugees and NGOs that operate on the field. This study aims at further understanding how Afghan refugees perceive and explain depression and largely psychological distress, and how this influences their choices for coping strategies and help-seeking.

**Methods:**

This qualitative study included six vignette-based semi-structured focus-group discussions with Afghan refugees and asylum-seekers (total *N* = 19, 12 female and 7 male) residing largely in camp settings in Northern Greece. The vignette describes a fictional person with symptoms of depression in line with DSM-5 and ICD-10 criteria. The interviews were recorded, transcribed, and analysed through template analysis.

**Results:**

EMs for depression are explained through Pre-migration Traumatic Experiences (PMTE) and Post-migration Living Difficulties (PMLD) relating to camp settings. Female participants identified gender-based and domestic violence as contributing to psychological distress while males highlighted conflict and persecution. Life in the camp with associated inactivity, and uncertainty for the future, was perceived as a significant risk factor for psychological distress among females and males. In terms of coping strategies, females tended to focus on mobilizing collective resources within the camp (e.g. safe space for women facilitating emotional support), while males advocated for self-empowerment and solution-oriented coping. The value of engagement in peer helper-roles was highlighted.

**Conclusions:**

The results highlight the potential value of community-based psychosocial approaches to support and promote mental health within camp settings. Additionally, they may inform policies and practices regarding access to appropriate mental health care for Afghan refugees. Further research is needed to establish the efficiency of such interventions in this context.

**Supplementary Information:**

The online version contains supplementary material available at 10.1186/s12888-023-04613-2.

## Background

In April 2021 there were around 6,6 million forcibly displaced people living in refugee camps worldwide [1]. With the war in Ukraine, the number is continually rising [2]. Lengthy administrative processes, complex political circumstances in host regions and continuing conflicts in countries of origin, often result in long-term stays in the camps, contributing to deteriorated mental health among inhabitants [3–6]. Aragona, Castaldo [7] argue that pre-migration traumatic experiences (PMTE) often interact with post migration living difficulties (PMLD), as is the precarious situation of camp life, further explaining psychological distress to a large extent, among refugee populations.

Health systems in camps must manage a wide range of mental, neurological and substance use problems [8]. While access to health services may often be limited, cultural variations in explanations of illnesses and preferred coping strategies and help-seeking paths may also constitute significant barriers to providing mental health services to refugees [9–11]. As Rasmussen, Katoni [12] point out, to treat, one must communicate, and to communicate well, one must understand the local explanatory models (EMs). The concept of EMs as developed by Kleinman, Eisenberg [13], provides a framework to address differences between health professionals and refugees, as well as between non-governmental organizations (NGOs) operating in the camps and beneficiaries. To date, studies of EMs among refugees in camp settings are very limited. In this study, we explore EMs and coping strategies among Afghan refugees, one of the largest groups of refugees living in camps in Greece. The major aim is to gain knowledge that may inform mental health services and relevant interventions in this context.

### Afghan asylum seekers and refugees

Nearly 6 million Afghans have been displaced from their homes, with nearly half of those fleeing to other countries [14]. High illiteracy, separation from economic and public life among women, gender-based violence, high impairment in daily life due to psychological distress, and lack of mental health services are some of the challenges the dominantly rural Afghan population faces [15–17].

Studies on EMs among Afghans in exile show that in addition to exposure to war and pre-migration trauma, perceived causes of stress include PMLD such as, acculturation, financial concerns, fractured social networks, negative social control, uncertainty about the future, and unemployment [9, 18–21]. Several studies report that uprooting and fractured social networks may influence traditional help-seeking paths, although there are conflicting findings using public mental health services. For instance, a study among Afghan refugees resettled in Australia and New Zealand found that few consulted health professionals [18]; while a study in Norway suggested more openness to utilizing public health services when experiencing mental health problems [19]. Gender and age divergencies are frequently reported in these studies, supporting the notion that EMs are multiple and influenced by life experiences. However, these studies may not necessarily be valid for inhabitants in refugee camps, whose physical environment and life situations may diverge strongly from refugees settled in their new home countries. Importantly, inhabitants in the camps are in a transit situation involving high uncertainty about the future as most are still awaiting the outcome of their asylum application. Previous studies have identified context-sensitivity and flexibility as key components of EMs [22, 23]. Against this backdrop we suggest that there is a need for more knowledge about how Afghans (and other refugee groups) perceive, explain, and prefer to cope with mental health problems when faced with the limited of opportunities of support inside refugee camps.

### Life in refugee camps; the situation in Greece

Arrival at a camp implies a slow adjustment process to a new and unfamiliar setting [24]. Dire living conditions, detention systems, lack of infrastructure and basic provisions, burnt out aid workers, uncertain futures, dispersed families, and loss of networks and social roles contribute to making life in refugee camps challenging [3, 25–27]. The life situation in the camps may influence refugees in profound ways. Cultural systems have been profoundly altered, including rituals, identities, social organization, and shared visions of the future, which, under other circumstances, may buffer the adverse impacts of stressful experiences [28]. Yet, studies have observed low refugee participation in decision-making processes, that concern their own situation, limited resources and conflicts between the diverse actors involved in providing services in the camps [29–31].

Since 2014, more than 1.2 million refugees have crossed into Greece [32], with almost 24 000 dead or missing in the Mediterranean [33]. Part of the response to this humanitarian emergency was the creation of camps in the islands and on mainland Greece with the potential to host large numbers of people. Such camps would often operate in repurposed military bases, former airports or even in makeshift accommodation spaces, often isolated geographically in rural areas [34]. Camps in Greece provide shelter in often unsafe conditions regularly exceeding the maximum capacity [3]. The result has been eruptions of conflicts, arson, deaths and deteriorated mental health among refugees in the camps [3, 29]. Additionally, the pandemic further impacted health and wellbeing due to lack of access to protective measures and increased restrictions [35]. The United Nations [36] have warned that the situation may exacerbate mental health problems among the refugees and limit access to the already scarcely available mental health services.

The Hellenic Ministry of Migration & Asylum [37] reports that as of November 2021, 28 accommodation sites are operating all over the country hosting more than 20.000 asylum seekers and refugees, while more than 170.000 refugees and asylum seekers were accommodated in the country [38]. Greece is primarily a transit country for displaced populations [39], but many remain stranded in Greece, disillusioned of the opportunity to travel further, and waiting for indefinite periods [40]. It is important to note that Afghan refugees often go through a lengthier asylum-seeking process, resulting in rejections and appeals that can stretch their transit state for years [39]. Data by the Hellenic Ministry of Migration & Asylum [41] report rejections of asylum applications reaching up to 50%.

The implementation of stricter policies has further exacerbated the ongoing situation in refugee camps. Organizations working in the field estimate that almost 60% of those residing in camps no longer have access to *“either sufficient or suitable food”*[42]. While the number of NGOs and INGOs operating in and around the camps was high until recently [29], the Greek authorities have currently enforced a registry for organizations eligible to work in the Greek state with refugees and particularly those residing in camps. As a result, the number of organizations eligible to provide support has become more limited.

Unsurprisingly, studies in Greek as well as in international settings, have found that refugee mental health problems are frequent among camps [3–6]. Additionally Braun-Lewensohn Abu-Kaf [43] document important gender differences for those living in Greek camp settings, highlighting the mediating role of social capital among refugee women. Thus, there is a need for *in-situ* studies focusing on how refugees in camps perceive and prefer to cope with mental health problems, as a starting point for the interaction and communication between health professionals and the refugees, as well as between NGOs and their beneficiaries.

### Explanatory Models (EMs) and coping strategies

Kleinman ([44] p105) defined EMs as “notions about an illness episode and the treatment employed by those involved in a clinical process, including patients, their families, and their doctors”^.^. Numerous studies have since shown cultural variations in EMs and argued that this knowledge is crucial to health promotion, utilization of health services, and ensuring culturally sensitive care and treatment compliance [45–48]. EMs have been included as a new addition in the Diagnostic and Statistical Manual of Mental Disorders (DSM-5) in the form of cultural concepts of distress [49]. Importantly, different EMs may involve preferences for coping with health problems outside the professional health system. For example, if the condition is interpreted as symptoms of spiritual factors, the person might be less likely to seek help from medical or psychological health services. In traditional Afghan communities [50], family and friends, as well as culture-specific healers and religious leaders have played important roles in diagnosis and decisions about preferred treatment options [9, 51]. War and displacement may disrupt familiar help and social support structures for refugees, leading to limitations and changes in how they approach mental health challenges [52].

Studies in this field have underpinned Kleinman’s original notion that EMs are dynamic and subject to change depending on the social context [19, 22, 53, 54]. EMs are not a priori cultural models but are based on life experiences in a globalized world introducing people to multiple and often contradictory explanatory models and sources of help and treatment [55–57]. Factors such as gender, age and education may influence EMs within cultural groups [19, 21]. For example, Alemi, Weller [21] observed that the EMs of Afghan women more than males, often centred on childcare responsibilities. Copolov and Knowles [58] found gender differences in coping strategies among Afghan population groups in Australia. Specifically, they argued that unaccompanied men separated from their families more often than females engaged in substance abuse to cope with their distress. In comparison, young women were more likely to cope through religion.

Pathophysiology, individual characteristics, and social responses can interact unpredictably, influencing how people understand and attempt to cope with their health problem [53]. The experience of shame and the perception of stigmatization in connection with mental health may be challenged by cultural integration processes in host countries in which such challenges may be more “normalized”. Positive encounters with professional mental health services in this new context may further influence the use of health services [19, 59].

In post-migration settings, specifically in camps, such changes are yet to be adequately documented. Given that various cultural groups often co-inhabit, knowledge about EMs among these groups is crucial for mental health professionals and other NGO workers inside these camps. Adjustment to the infrastructure of camp life, lack of traditional coping resources and options, and exposure to different cultural views about the causes of and treatment of mental health problems may influence refugee EMs in fundamental ways.

### Aims of the study

Based on the context-sensitive nature of EMs, how they affect coping strategies, and the lack of studies about EMs in refugee camps, the present study aims to better understand EMs among Afghan refugees in Greek camp settings. By this research, we intend to provide knowledge of applied value for health professional, NGOs, and policy makers in providing targeted services for this group.

## Method and materials

### Design

This is a vignette-based focus group study. Focus groups were used, as it allows for discussions among the participants, revealing possible ambivalence and diversity in explanations. The vignette technique has been considered optimal in qualitative research on themes that might bring up difficult emotions or may be associated with stigma among vulnerable populations [19, 47, 60]. Vignette-based focus groups might provide the safety of distance when exploring sensitive subjects and facilitate sharing of views from a non-personal perspective [61]. The vignette used in the present study, described a fictional person, gender matched to the respondents, experiencing symptoms of moderate depression in line with the criteria of DSM-5 and ICD-10. The vignette was designed to display a refugee/asylum seeker living in a camp setting in Greece with her/his children (table S1).

### Interview guide

The vignette was introduced at the start of the interviews, which were semi-structured following an interview guide (table S2) adapted from past research [47] and building on Kleinman’s ethnographic approach [44]. It was pilot tested with two Afghan interpreters.

The moderators encouraged open discussions, attempting to involve all participants, ensuring that the communication was focused, and that all topics were covered. Exploratory open and closed questions were introduced to facilitate discussion and allow spontaneous feedback from the participants.

### Participants

The sample consisted of adult Afghan asylum seekers and refugees. The inclusion criteria for participating in the study were: 20 – 50 years of age, having a refugee or an asylum seeker status, speaking Dari or Farsi, and having an Afghan ethnic origin. These criteria were set, based on the demographic profile of the Afghan refugees and asylum seekers. The majority are young adults or individuals up below the age of 50. Also, the age criterion used in the study was also relevant to the experience of being a parent which was integrated in the vignette. According to UNHCR [62], a *refugee* refers to a person legally recognized as needing international protection according to the criteria set in the 1951 Refugee Convention while an *asylum seeker* is a person seeking such protection status according to Article 14 of the Universal Declaration of Human Rights. However, in the present paper, we use the term *refugee* as an umbrella term independent of the legal status.

The sample consisted of nineteen individuals. Data was collected from six focus group interviews divided by gender (male and female). Demographic information is presented in Table 1. Aside from three males residing in a nearby village with recent experience from living in camps, all participants lived in camp settings in Northern Greece at the time of the data collection.

**Table 1:** Demographics

	Gender			
	Female (*n* = 12)		Male (*n* = 7)	
Age (range)	20–25 yrs		20–25 yrs	3
	25–30 yrs		25–30 yrs	2
	30–35 yrs	7	30–35 yrs	1
	35–40 yrs	4	35–40 yrs	
	40–45 yrs		40–45 yrs	1
	45–50 yrs	1	45–50 yrs	
Origin	Afghanistan (12)		Afghanistan	6
			Iran	1
Status	Asylum seeker	10	Asylum seeker	3
	Refugee	2	Refugee	4
Time in Greece (range)	0–1 years	1	0–1 years	1
	1–2 years	6	1–2 years	1
	2–3 years	3	2–3 years	2
	3 + years	2	3 + years	3
Marital status	Married	9	Married	2
	Single		Single	5
	Widow	2	Widow	
	Other (Divorced)	1	Other (Divorced)	

### Procedure

The vignette was designed by the first author based on his clinical experience working with refugees in Greek camp settings. The full vignette is presented in the appendix. The vignette and interview guide were translated from English to Dari by an official translation agency followed by a back-translation by a bi-lingual Afghan cultural mediator.

The data collection took place from July 2021 to November 2021 until saturation was reached. Due to pandemic restrictions, four focus groups were conducted online or through a hybrid mode. In the two online interviews, the participants, the moderator, and the interpreter were located in different places. In the two hybrid interviews, all participants were in the same room while the moderator and the interpreter participated via distance. Two interviews were conducted physically since the Covid-19 health regulations allowed it at the time, taking necessary precautions (i.e., face masks, open windows). Except for the latter interview moderated by a male (author 1) and a female researcher (author 3), the moderator was a male (author 1). Some female participants brought their children along for the interview. In the cases where participants spoke in Dari, the interpreter translated into English. Among three interpreters involved in the present study, two had an Afghan refugee background and were unknown to participants. The third had Iranian migrant background and had met the participants before as a humanitarian worker. More detailed information is presented in Table S3 in the supplementary material. The participants were recruited through humanitarian organizations working with the target population. The humanitarian workers handed out a short notice explaining the research in light language and clarifying that no extra help nor restrictions would come from the organization in case of participation or refusal to do so. Those who expressed interest gave their consent to be contacted about the interview to the coordinator of the local team. Small data packages were given to the three participants living outside the camps to facilitate their participation. The remaining participants used the organizations’ resources and did not receive any compensation. All who expressed interest to participate and met the inclusion criteria were invited to the focus groups.

Before the interviews, participants received information about the study and their rights as research participants and signed an informed consent form in Dari. All but one agreed to participate in the study. The participants either read the consent form themselves or it was read aloud by the interpreter. There was time for questions and answers to ensure that the participants understood the information provided. In digital interviews, signed consent forms were locked in the office of the organizations until given to the first author.

The interviews lasted between 50 to 90 min. Online and hybrid interviews were video- and audio-recorded while physical interviews were only audio-recorded. No one else but the participants, the moderators and interpreter were present during the interview. Due to language barriers participants did not provide further comment on the transcripts or feedback on the findings.

#### Ethics statement

Approval for the data collection was first sought by the Norwegian Regional Ethical Committee which stated that since no health data would be collected, it was exempted from their scope. The Norwegian Center for Research Data (Reference number: 119451) approved the study. A crucial matter relates to handling participants’ distress while listening to or discussing potentially traumatic events. In these cases the interviewers who have extended clinical experience in their role as psychologists applied psychological first aid skills. Two were followed up with psychosocial care and the support of the cultural mediator and humanitarian organizations.

### Analysis

The first author and a research assistant first transcribed the interviews verbatim into English. The transcribed data was anonymized by removing any potential information that could lead to the identification of participants. Audio- and video-files were digitalized and kept safely. After transcription, the original files were deleted.

Working with the data, we followed template analysis [63]. All authors first familiarized ourselves with the data separately, going over the transcripts of the first four interviews. Then we jointly agreed on categories related to the data in a preliminary data coding phase. This was followed by clustering the themes under the categories and identifying the relation between each cluster. Comparing findings, an initial coding template was formulated. Then, each author separately applied this template to the two remaining interviews. Then, the template was adjusted, and it was applied to the full data set of the interviews, making final modifications to the template helping form a map of the different clusters under each category. Feedback on the coding categories was provided by a reference group consisting of people with lived experience in forced migration originating from Afghanistan and in Greece at the time. NVivo12 software was used to code the transcripts and identify each cluster’s density.

## Results

Findings were grouped into two main categories: explanatory models of depression; and coping strategies. Constructed themes are presented under each category, separated by gender and the overview of main themes and categories are presented at Tables 2 and 3. Most participants, both females and males, identified with the vignette character expecting that any person experiencing similar adversities and life challenges would have similar symptoms. A male participant indicated that “the stress would be normal, because any person in this situation will have stress” (M1,^1^ [5]^2^). Hearing the story of the vignette character, many talked about a similar experience. One participant stated that “all Afghan women have the same problems as Adela [the vignette character].” (F4, [3]). Some explicitly used the term depression: “They [women in the camp] have depression, mental health [problems][…] they worry about the children, they worry about the [asylum] interview in this country” (F5, [3]).

**Table 2:** Central themes in female focus groups

Main Category	Theme	Illustrative quote	Number of participants
Explanatory Models of depression	Pre-migration Traumatic Experiences: Perpetual gender-based violence	In Afghanistan it is kind of like a man-ruling country. It is not for women. Everything, every right has gone for the men. F3	3 (FG1) 1 (FG2) 5 (FG3)
	Post-migration Living Difficulties: Life in the camp	It’s a really difficult situation that we are living in the camp. Of course, you get depression in this situation. F1	2 (FG1) 2 (FG2) 4 (FG3)
Coping Strategies	Social Support	You feel ‘ok there are more women that have the same problem’. This makes you feel better and stronger to continue. F4	3 (FG1) 3 (FG2) 4 (FG3)
	Cognitive Strategies	The future about us and the future of our children. Think about the progress. F4	3 (FG1) 3 (FG2) 4 (FG3)
	Professional Help	The women here are, basically all of them, under the psychiatric care here. I am going to the psychologist for three years and one month now. F12	1 (FG1) 1 (FG2) 1 (FG3)

**Table 3:** Central themes in male focus groups

Main Category	Theme	Illustrative quote	Number of participants
Explanatory Models of depression	Pre-migration Traumatic Experiences: Exposed to violence in Afghanistan	“I am driving the car on the street and you [hear] explosions in this or that area. Always stress, always fighting. M7	2 (FG3)
	Post-migration Living Difficulties: Life in the camp	In a camp, especially in the camp that we are living, with high walls around the camp, [it] is like a prison here. Every night happens something new to the people. So, this is actually bringing stress. M2	2 (FG1) 3 (FG2) 1 (FG3)
Coping Strategies	Social Support	They were volunteers and now they have life, they are married. M4	1 (FG1)
	Cognitive Strategies	If you want to change the way, I mean a change to Adel [vignette character], you have to be strong, you have to give him the motivation to change his mind. M5	3 (FG2) 2 (FG3)
	Solution-oriented coping	We can tell him, there’s a place that you can go for 3–4 months to improve your speaking skills and you make a CV, cover letter and also you send your kids to the kindergarten […] Then you find a job, then your life will be much better. M3	1 (FG1) 3 (FG2)
	Professional Help	Having a psychologist and having a lawyer to inform him daily for the procedure and his documents. These two are the solution to his problems. M1	2 (FG1) 3 (FG2)

### Explanatory models of depression

Participants attributed depression to adversity, before and during flight and the trials of life in the refugee camps, which further aggravated burden of past experiences. Females emphasized gender-based violence and the role of women in Afghan society, while males stressed experiencing violent conflicts.

#### Females: Pre-migration traumatic experiences; perpetual gender-based violence

Being a woman in Afghanistan was highlighted as a risk factor for psychological distress since Afghanistan “is not safe for women” (F3, [3]) and considered a “man-ruling country” where “every right has gone to the men” (F3, [4]). Several participants further commented that there are few or no opportunities for women in work and education: “women cannot go out, cannot study” (F9, [4]). The situation in Afghanistan was mentioned as the most important reason for seeking exile. One participant stated: “If we were relaxed and safe there, we wouldn’t go anywhere! We would have stayed there and lived there” (F7, [4]). Child and forced marriage were frequently mentioned: “And when I was 13 my father married me to somebody. And what they do, is they raise their daughters until they are 13” (F12, [4]). Participants discussed facing gender-based violence in many forms and how attempts to flee or fight in abusive relationships were followed by further abuse. One explained:

*“I told my father “Why, why did you marry me? I didn’t want to.” And he started hitting me, so much. Every time I felt like complaining again, I was in a very difficult situation, I would remember what happened the last time I spoke with my father, and I would just… stay… silent”. (F12, *[4]*)*

Several participants had crossed into Iran with their families, where they faced further struggles and discrimination:

*“We had no rights, that is what led us to, you know, [to] migrate”, and “when I was in school studying, in Iran and the head mistress came into the classroom, she would open the door and say: “Afghani, come out”. When we were doing the daily prayers, they would ask the Afghani to go to the back of the class. They did not count us as human beings”. (F8, *[3]*)*

Others recalled life threatening situations during on the way to Europe:

*“When we were on the boat we fell in the water, and we were in the water for half an hour. And […] we are not very well mentally. So, my other kids are fine, but the one 6-year-old I have now… I think it’s because of the water incident… she is kind of not well. And I am also not well psychologically. I think maybe the shock that was given to her, you know, during falling in the water, has made her this way”. (F11, *[4]*)*

#### Females: Post-migration living difficulties; life in the camp

Females acknowledged feeling safer in the camp than in Afghanistan. Yet, living in the camp involved other stressors such as uncertainty and inactivity. One commented: “It’s a really difficult situation that we are living in the camp. Of course, you get depression in this situation” (F1, [3]). Another described a feeling of deep sadness that she tried to conceal living in the camp; “I walk through the camp looking very happy. But inside, I am not. Because my family is living in a very terrible situation” (F9, [4]). Several talked about carrying a huge burden, “The problems here are not just one or two. It’s very difficult. I’m sorry. It’s very difficult” (F9, [4]).

##### Uncertainty

Uncertainty is widely prevalent in camp settings. In our study, uncertainty for the future was emphasized as a major source of distress. One commented, “We don’t know about our own future, what will happen to us. What can we recommend to her? [the vignette character]” (F1, [3]). Another participant described the lack of stability and predictability of being without a permanent place to live for themselves and their children: “We need to say that we have a home, and we know that it is somewhat stable. That we can stay. We are tired of, you know, coming and going and not knowing our situation” (F11, [4]). They also worried about their children’s future, “I have a newborn baby and I think a lot about her future. I am thinking about her future. Can I register her to school? What will happen to her?” (F3, [3]).

Talking about causes of mental health problems, participants stressed the restrictive refugee policies and many expressed hopelessness about the future:

*“Everyone kept telling us that the people who had been rejected once, would definitely be rejected again for the second time. And we were very hopeless. And the fact is that yes, we also got rejected the second time. I was crying all the time. I was completely hopeless. I was worrying about my children. I also gave a third interview, but I did not have any hope at all.”. (F7, *[4]*)*

Rejection of the asylum application could potentially cause separation of families: “We have got accepted, [but] one of my children has received a rejection”. (F10, [6]).

##### Cycle of Inactivity

The issue of having nothing to do in the camp was frequently raised among the causes of mental health problems: “Here there is absolutely nothing. No classes, nothing for women” (F8, [3]); “we are just, you know, in our own thoughts. Just thinking” (F9, [4]). Inactivity contributed to lowered mood which in turn impacted their experience of coping with parenting and aggravated psychological distress: “Sometimes my children come and ask me “What happened to you?” I say, don’t talk to me, I don’t want to listen to you, I want to be silent” (F1, [3]). Another stated: “I was crying all the time. I was completely hopeless. I was worrying about my children” (F1, [3]). Participants stated that they felt locked up in this cycle. One explained that: “I cannot help myself when it gets worse” (F3, [3]), while another pointed out that “everything is more and more difficult” (F1, [3]).

#### Males: Pre-Migration Traumatic Experiences (PMTE); Exposure to violence in Afghanistan

Participants emphasized that the violent conflicts in Afghanistan contributed to the high levels of distress. Several talked about significant distress connected to witnessing violence, murder and atrocities in their childhood and later in life. One recalled,

*“One day I was coming back from school […] There was a water source and I went to drink some water. I went alone. There I saw a person whose head was cut [off]. I was only ten years old. I saw cut [them] like that [explains how his neck was cut]. After that my family took me. Everyone from my family came to take me. I had my eyes open all the time at the house. After that, for six or seven months, I didn’t get out.” (M6, *[2]*)*

Another recalled, “I am driving the car on the street and you [hear] explosions in this or that area. Always stress, always fighting” (M7, [3]).

##### Taliban takeover

One of the male interviews took place shortly after Taliban returned to power in Afghanistan in August 2021. The two participants discussed how they followed the events of the violent regime change in, closely through social media. Both talked about distress associated with thinking about the aggravated situation of people in their home country. One commented, “Two days ago, the Taliban attacked a boy and killed him because he was wearing headphones and was listening to music. I have the videos” (M7, [3]). The same participant described how the current regime would retaliate against accused terrorists who worked with the previous government: “You are a terrorist because these people say so. Then they arrest you and you might get lashed. I have videos” (M7, [3]). Male participants went on to further explain that they might have had better living conditions in Afghanistan. Still the continuous armed conflicts, led to their decision to leave the country as the abovementioned participant further emphasized, “In Afghanistan you can have a car, big house, job, but feeling like that in this country is not better day by day. It’s always fighting in this country. Taliban might attack your house and family” (M7, [3]).

#### Males: Post Migration Living Difficulties (PMLD); Life in the camp

The camp was often described as a crowded place of confinement, isolation, and unrest: “In a camp, especially in the camp that we are living, with high walls around the camp, [it] is like a prison here. Every night happens something new to the people. So, this is actually bringing stress” (M2, [1]). One stated that “people from many countries are living in the same camp. And they are sleeping every night of fear that maybe something [will] happens to them” (M1, [5]). Participants attributed aggression to “psychological problems”: “some of them were really getting angry in different situations. They wanted to fight, they wanted to make problem” (M2, [1]).

Lack of meaningful activities and detachment were also discussed as relevant to life in the camp: “Every day it was the same. I didn’t have anything to do. There was no motivation to do something” (M2, [1]), while further addressing passivity “No motivation. You just make yourself sad, you don’t want to get out or communicate with others” (M2, [1]).

##### The burden of family obligations

Feelings of distress were closely linked to concerns about their children in the camp:

*“The situation in the camp; [there is] no good food, you don’t get good money for a living, [while] you have three sons. The sons want a better school, better area, better life, better [inaudible]. The father is always looking at his sons’ faces. If the babies have no good life the father also has no good life.” (M7, *[3]*)*

The discussions related to the vignette focused on his family situation. One suggested “At least for his [the vignette character’s] children, he needs to make life really well. Now they are young, but in 2–3 or 5 years they will grow up and it’s super difficult, but he has to support them” (M4, [2], male). They emphasized that the loss of [the vignette character’s] his wife further family responsibilities for the husband: “He lost his wife, and all her responsibilities fall on him. He needs to take care of everything” (M3, [1], male). Managing family everyday life while dealing with the stress of the upcoming interview is difficult: “if you have three children that don’t go to school and you are also waiting for an [asylum] interview, it’s a big problem”. (M7, [3]).

##### Uncertainty

Many refugees considered Greece as a temporary stop on their way further into Europe. However, the lengthy legal process and the limited opportunities to move on from Greece were associated with increased distress. One explained that “all of them are thinking about one option: positive result from the asylum interview. To be able to leave this country, to go somewhere else and make a new life over there” (M1, [5]). Thus, the outcome of the asylum interview was tightly linked with hope for the future or feeling deadlocked: “They are afraid if they have a negative result from their asylum interview, then they would not be able to go back to their own country. And they will not be able to go to the other countries. And they will not have enough money to move illegally” (M1, [5]). Being in an uncertain situation brings up feelings of distress: “Deportation and thinking about everything, it really makes you feel sad, because you don’t know what you are going to do tomorrow” (M4, [2]). Another added that “Waiting for a decision, waiting for ID, waiting for passport… You are always wondering ‘What about me? What about my life? What about my future?’. This creates stress”. (M7, [3]).

##### Cycle of inactivity

The participants described a vicious cycle of demotivation and social withdrawal. One states, “I was inside. I didn’t go to classes for the languages. I didn’t meet anyone else. For 6 months it was so difficult for me. I couldn’t talk to anyone […] I was depressed” (M4, [2]). Perceived inability to take care of themselves and their children in turn aggravated the feeling of distress: “a person who has this kind of mental and psychological pressure, it’s normal not to be able to take care of his kids and himself” (M2, [1]). Sense of losing control on a cognitive level was part of this cycle: “when you are depressed, you cannot control your thinking” (M2, [1]). To some, this involved rumination about life: “I was thinking about my life too much, past and future but without any reason” (M4, [2], male).

### Coping strategies

The escape from the war and adversities in Afghanistan also implied leaving important coping resources behind, extended family members, neighbors, religious counseling, village meeting points, and where available, Afghan psychologists. One stated, “In our own country the situation would also be different because we would have our friends, relatives, family that would not let us be in that situation” (M2, [1]).

Another, relating to Afghans living in Iran, stated, “In Iran you have your parents they can help you, try to understand you and make life easier for you” (F2, [3]).

#### Females: Social support

Given that distress was seen as a shared experience among refugees in the camp many emphasized coping with the situation collectively: “You feel ‘ok there are more women that have the same problem’. This makes you feel better and stronger to continue” (F4, [3]). Community relationships formed in the camps provided social support and distractions, “When I am really, really stressed out psychologically, I will prefer to leave the house… the container… and go and see some friends. Just to laugh, and just to talk about something” (F8, [3]).

Seeking support from remaining family inside and outside the camp was emphasized, “When you have family, you kind of feel that you have support. You kind of share the problems with each other” (F8, [3]). Social media and cell phones enabled contact with family members outside Greece. However, while the women needed this kind of support, they did not want to cause distress for distant family members by revealing their bad situation in the camp. Not sharing alarming or distressing news was a way to protect their distant support network: “Sometimes when my mother calls, I am forced to lie about my situation here. Because if I tell her the truth, she is going to be very worried” (F8, [3]). At the same time, some participants emphasized that family support would not be sufficient for relieving the distress they experienced, “I don’t think that family can help. It’s more like showing sympathy” (F1, [3], female).

#### Females: Cognitive coping; hoping for the future

Hope was a central theme across the focus groups. First, they expressed the hope that difficulties are only temporary. One argued: “We say to ourselves: ‘This shall pass’ and ‘Things shall get better’. And since we all have, you know, psychological problems, this is the sentence we use” (F8, [3]).

Second, hope centred on a better and safer future in Europe, away from the struggles in Afghanistan, e.g., one mentioned: “They understand that here is Europe, women have rights, and everyone knows about the rights. It will get better and better”.: “Just think about Afghanistan, there’s war, there’s no security, at least here we are safe with our children” (F4, [3]). A third perspective of hope, focused on children’s wellbeing and future opportunities for development: “The future about us and the future of our children. Think about the progress. The children being a doctor, an engineer having a bright life and a bright future. This makes us feel confident” (F4, [3]). Finally, the interest from outsiders such as volunteers and researchers provided a sense of hope for the future: “A hope is planted inside the heart of the Afghan people, when we see that people from different countries actually care about us” (F8, [3]).

#### Females: Professional help

Participants prioritized seeking help within their community when experiencing distress. Yet, professional help was also available in the camp and in some cases in cities nearby. Some shared their experience seeing a psychologist when this was considered the only option:

“Participant: The women here are, basically all of them, under the psychiatric care here. I am going to the psychologist for three years and one month now […]

Interviewer: That’s very good to hear that you have got help and that you…

Participant: Yes, there is no other solution”. (F12, [4]).

However, there seemed to be ambivalence regarding the efficacy of psychological treatment. One stated, “I went to the psychologist for three months, but I did not get anything good out of it”. Later, explaining how she didn’t receive the help she was looking for, she stated “I really don’t know what could have helped me. And the psychologist wasn’t helping me because she kept saying ‘cry, cry’” (F11, [4]). The assumption that health professionals lack understanding of Afghan culture, history and society was seen a further barrier: “Here in Europe, many psychologists cannot understand us. And they don’t have any solution to give us. […] But in Afghanistan they know our problem and it’s easy to find a solution for us” (F4, [3]). Further explaining her argument, she stressed that health professionals might lack knowledge about the specific situation of women in Afghanistan: “That’s why I say they have no idea about our problems” (F4, [3]).

#### Males: Social support

Usually when living in Afghanistan, participants would seek help from their social and family networks which might not be available in their current situation: “In our own country the situation would also be different because we would have our friends, relatives, family that would not let us be in that situation” (M2, [1]). To remedy the situation for themselves, some participants suggested taking up a helper role towards other refugees and asylum seekers in the camps “For such a person in Greece I would suggest engaging himself being a volunteer […]” (M2, [1]).

##### Getting together and taking up a helper role

To the participants’ perspective, being a helper could improve meaningfulness in everyday life and contribute to future opportunities: “Like you see a person living in a refugee camp and working as an interpreter. You will see he is improving, doing lots of things and it really helps you. You see the person working, having a life, coming back” (M3, [1]).

Becoming formally engaged with NGOs in the camps was described as a way to expand social networks and as a possible gateway to employment: “After a while I went to [name of organization] and I met so many refugees. They were my colleagues. Right now, they have left, they have jobs, some of them left to another country” (M4, [2]). The same person further proposed that engagement in activities could even lead to finding a partner “They were volunteers and now they have life, they are married.” (M4, [2]). Thus, taking on a helper role embodied prospects of a better future after leaving the camp in several ways. Reflecting on his own experience as a volunteer, the same participant stated, “My job is my hope”. (M4, [2]).

##### Responding to parental obligations

While the males considered the burden of family obligations in the setting of camp life a source of distress, they emphasized that the responsibilities that come with fatherhood could also be a coping resource.: “The important thing is that he has to be strong because of the children. The children are going to look up to their father, to what he is doing. They are going to learn, learn from the father” (M5, [1]). Since the vignette character was described as a single parent, the importance of finding a wife was emphasized: “Maybe he needs to have someone to take care of his children. […]. He might need to find a partner to make it easy for him” (M3, [1]).

#### Males: Cognitive coping

Cognitive mechanisms such as reframing was suggested as an essential way of relieving distress: “If you want to change the way, I mean a change to Adel [vignette character], you have to be strong, you have to give him the motivation to change his mind” (M5, [1]). Other participants emphasized the need for mindfulness of the present and for visualizing the future: “You center yourself in the present” (M3, [1], male); and “There have been a lot of problems, a lot of bad situations. But it was in the past. But now the tomorrow is your future” (M5, [1]). Trying to overcome what they sometimes perceived as an impossible situation, several talked about coping by forcing themselves to take control: “It belongs to a person to decide what they want. Do they want to get out of the situation or not?” (M2, [1]).

#### Males: Solution-oriented coping

Thinking should eventually lead to action through small steps. One said:

*“I started thinking I cannot continue with this. I have to change my life. I cannot be in my container all the time. I have to move, I have to change my life. If this situation continues, then I cannot enjoy my life. But step by step, I am happy I did it”. (M4, *[2]*)*

Another participant suggested that “he [the vignette character] can start slowly, slowly to do some activities, to do something better, to feel better” (M2, [1]). Practical solutions and steps that can lead to a sense of empowerment were emphasized: “We can tell him, there’s a place that you can go for 3–4 months to improve your speaking skills and you make a CV, cover letter and also you send your kids to the kindergarten […] Then you find a job, then your life will be much better” (M3, [1]). Getting active to manage psychological distress was further highlighted: “If you don’t do that and stay at your container, or home anywhere. You will be dead” (M3, [1]).

Thus, the males emphasized that the main responsibility lies on themselves to improve their situation. Interestingly, participants emphasized that even God would not solve their problems for you: “Well naturally when people have no other option, they try to refer themselves to God. Now I want to share my personal belief that God will never interfere with peoples’ life. I mean if you pray now for tomorrow, God will not do anything for you if you do not move for yourself.” (M1, [5]).

#### Males: Professional help

Participants did not seem hesitant to seek professional help. For example, they advised the vignette character to talk to a psychologist and a lawyer involved in procedures around asylum seeking. One stated, “the solution for this person is twofold; Having a psychologist and having a lawyer to inform him daily for the procedure and his documents. These two are the solution to his problems.” (M1, [5]).

## Discussion

In line with previous studies [22, 23, 44], we identified context-sensitivity and flexibility as key components of EMs, and investigated for the first time qualitatively, how a group of refugees perceives, explains, and prefers to cope with mental health problems when faced with the limited opportunities inside refugee camps. Our findings mirrored previous studies in refugee camps that describe PMLD; lack of infrastructure and basic provisions, uncertain futures, worries about the ability to care for their children and the future of their children, dispersed families, lack of control and loss of motivation, violent conflicts, and loss of networks – but also new relationships within and between cultural groups, improved rights for women, and opportunities through NGOs, INGOs and the UNHCR [8, 25–27, 29]. Participants explained the symptoms of the vignette character as results of gender-based adversities and the violent conflict in Afghanistan, but also from the situation in the camps. They identified strongly with the symptoms of the vignette character, expressing that such reactions are common among refugees in the camp. Several studies have further documented the increased prevalence of psychological distress and mental health problems among refugees in camps e.g. [3–6]. Importantly, depression and PTSD were not seen as medical issues, rather, the participants highlighted the normality of their reactions given the adversities they had experienced before, during and after the flight.

Consistent with coping literature [64], the data point towards a dynamic and reciprocal relationship between hope, engagement in coping strategies and emotional wellbeing; each in turn supports and is supported by the other. All the interviews emphasized that the waxing and waning of hope were contingent on news about the asylum application processes. This is in line with research [65] that documents how applying for asylum relates to increased distress where individuals have to support their claim in an unfamiliar and often unwelcoming system. Pandemic restrictions increased bureaucratic challenges for asylum seekers who had to be interviewed remotely to a large extent [66]. Loss of social networks and the physical and social conditions in the camp made it difficult to manage psychological challenges in the way they preferred and had been used to. The loss of coping resources and changed life situation contributed to participants emphasizing differences between what they would have done to cope with the situation back in Afghanistan, and what was possible within the camps in Greece. For instance, all focus groups emphasized the importance of informal emotional and social support when experiencing distress. This aligns with previous research on Afghan refugees in other contexts [19]. Some expressed reluctance to talk about their problems with others in the camp or with family in Afghanistan, who experienced distressing life situations like themselves. Thus, they experienced difficulties using the normally supportive links with extended family and community members.

There were some noticeable differences between females and males. Females tended to attribute the symptoms of the vignette character more often to gender-based and domestic violence. They highlighted their experience of marrying and giving birth at young ages, and of physical abuse within the family. That is congruent with their long experience of adversities, even while in exile in neighbouring countries, such as Iran [67]. Males tended to focus more on war- and persecution-related trauma.

Women used cognitive restructuring to facilitate hope during their stay in the camps. Hope for themselves, meant hope for their children as well. Braun-Lewensohn, Abu-Kaf [43] argue that refugee women in Greek camps often experienced a “healing effect” in time. Even though, this was not the case for this study’s participants, several women acknowledged a feeling of safety in Greece. That also was part of their cognitive coping strategies, comparing how the situation was in their home country to their current situation in Greece.

Participants sought help through different sources. In line with previous research [68] the interviews with females showed scepticism toward mental health professionals unfamiliar with their background. This might illuminate recent research showing that healthcare service utilization rates among refugees in camps are particularly low for common mental disorders [11]. Nonetheless, more females than males mentioned mental health care services as help seeking source for psychological distress. This is consistent with studies drawn from refugee populations [11, 69, 70]. Social support among women resembled what was previously described as emotional support involving meaningful interaction with each other. While the males were not opposed to seeking social support or even professional help from psychologists and human rights lawyers, they emphasized ways to empower themselves to cope with the situation. This involved becoming a peer helper and exploring other possibilities through NGOs and INGOs, taking on parental obligations, and reframing cognitive patterns to slowly become more active and capable of solving problems and regaining hope for the future.

The EMs of these Afghan refugees, then, were context-sensitive and flexible in their character, reflecting objective socio-political factors and economic and social constraints and facilitating factors such as the availability of coping resources within the camps, as much as underlying subjective cultural models [56, 71]. While our findings reflect gender-related cultural aspects of Afghan cultures such as the subordination of women and the emphasis on male strength in a patriarchal structure, practical concerns related to their vulnerable situation inside the camps came to the forefront of the EMs. The vulnerability of refugee women in camp settings has been reflected in international studies highlighting the risk of women becoming victims of gender-based violence and human trafficking [72–74]. As Silove and Ventevogel [16] argue, women in Afghanistan have lived through significant inequalities that excluded them from education, employment, and other essential aspects of life. Whatever progress was also achieved during the last years is being set back after the Taliban takeover of Afghanistan. EMs also changed with the political events in Afghanistan. Specifically, in both interviews, after Taliban returned to power in August 2021 and November 2021, participants discussed emotional suffering related to concerns about the current situation in their home country. Taken together, the most salient explanatory model of the participants highlighted environmental adversities as the main causes of psychological symptoms. It is worth noting though, that sometimes it was difficult to discern explanatory models from symptoms of mental health problems among the participants. For instance, the feeling of hopelessness among some women and the sentiment that neither professional help nor social support will help might reflect symptoms of depression, as much as any underlying EM about illness. Also, one woman mentioned that she quit psychological treatment as she disagreed with the therapist in views about the value of crying. Her reluctance to cry in front of the therapist may reflect emotional avoidance, a core symptom of PTSD. However, this situation could also emerge from cultural differences in norms about emotional expressions.

Living in a confined state, due to the structure of the camp as well as the geographic remoteness, offering poor stimuli and increasing the sense of exclusion from society can be associated with a sense of apathy and loss of motivation in what was termed as “institutionalism” by Wing [75]. In this study, living in the camp often explained the feeling of emptiness among refugees. Pérez-Sales, Galán-Santamarina [76] similarly identified extreme levels of disinterestedness and apathy among refugees residing in Moria camp and attributed such findings to the harsh living conditions. Pūras [89] identifies the socio-economic and cultural determinants as significant risk factors for mental illness and associates the right to health with the right to live an independent life in the community.

Notably, religion or spiritual explanations were hardly discussed. While this may partly be due to a lack of willingness to discuss these aspects with European researchers with possible prejudices against Islam (Brea Larios et al., 2022), the participants emphasized that although they may find consolation in prayer, even God will not solve their problems. This is congruent to the Islamic emphasis on individual responsibility and self-determination [77, 78].

### Limitations

The study has limitations. First, Afghan body language and culturally relevant expressions might not always be understood to their full extent by the researchers due to language and cultural barriers. A short discussion between the interviewers and interpreter followed the interviews to clarify potential misconceptions. Two Afghan language experts were also consulted to provide insight into the collected material. Second, there may be a gender dimension at play in the interview situation in the two focus-groups with female participants moderated only by a male researcher. To remedy this, the last female focus group was conducted with a female researcher participating alongside a female interpreter and a male researcher. Third, the gender of the interpreter and the translation, might contribute to further misunderstanding and inaccuracies. For that reason, in several instances, we employed an external translator to make sure that data was accurately translated. Additionally to gender, a bias could be attributed to the Iranian origin of one of the interpreters who also was a humanitarian aid worker. The choice to work with this interpreter was made, based on her trust relationships with the population and the fact that she was not working directly with them anymore at the time of the interview. Finally, Covid-19 crisis might have exacerbated the feeling of isolation for these refugees and is likely to have influenced the study results. The pandemic also resulted in variations in how the interviews were conducted, online, hybrid, or face-to-face. While this flexibility facilitated the inclusion of participants, it might have resulted in variability in the report between researchers and participants, access to non-verbal cues, and interaction between participants. Also, the researchers had less control over the location of the hybrid and digital interviews [79]. It should also be noted that refugees who participated in this research might not be representative for Afghan refugees in the camps. It should also be stated that there were more female than male participants. Male participants were in average younger than female participants. These differences should be kept in mind when interpreting the results. Taking part in the interviews might itself reflect an attempt to cope with psychological challenges as this might be seen as a chance to reach out to the international community about their situation.

## Conclusions and future research

An important finding from this study is the view of the refugees that mental health problems are normal and common reactions to extreme experiences and expected to improve through social and emotional support. If individuals do not view common mental health problems as medical issues, they may be less likely to seek care through formal health services and more likely to turn towards informal psychosocial supports within the community [10]. This is in line with findings from our interviews and highlights the potential value of non-medicalized, community-based intervention. As suggested by the participants, empowerment is central to the ability of refugees to withstand the significant stressors experienced in the camps.

There are significant recommendations that can be derived through this study. First, psychoeducation in appropriate language can help refugees understand and cope with distressing thoughts, emotions, and psychosomatic symptoms. Providing brief and practical information can further reduce barriers of mental health service utilization. Second, there is a growing body of evidence that competence-building activities initiated by the refugees themselves [80] or engagement of refugees in formal helper roles within NGOs [81–83] might promote mental health and psychosocial wellbeing. Introducing peer-led psychological treatments in such settings is continually proven effective in addressing treatment gap in depression and psychological distress even in high-income context [84]. Belonging in the family of interventions aiming to scale up mental health care in primary care context, the World Health Organization [85] developed Problem Management Plus (PM +) as a manualized response to a wide range of mental health problems [86]. So far, it has been implemented in European context and specifically for Afghan refugees, as it is already being documented in Austria [87] and Greece [88].

Conclusively, scaling up mental health care in such ways, involving collaboration between different actors on the field, will increase access to culturally appropriate mental health care for refugees facing adversity.

However, to establish best practices, more longitudinal studies are needed. Researchers and mental health care providers should recognize possible gender differences in experiences and coping resources within the Afghan refugee population and that trauma among females might be closely related to domestic violence.

## Supplementary Information

**Additional file 1.**

## Data Availability

Data collected in this study is stored at a secure server at the University of Bergen (called “SAFE”). Due to sensitive data relating to A population at risk, it is not publicly available. Data is available upon reasonable request to the corresponding author (michail.lavdas@uib.no).

## Abbreviations

DSM-5: Diagnostic and Statistical Manual of Mental Disorders 5
EMs: Explanatory models
FG: Focus group
ICD-10: International Classification of Diseases 10
INGOs: International non-governmental organizations
NGOs: Non-governmental organizations
PM +: Problem Management Plus
PMLD: Post-migration living difficulties
PTSD: Post Traumatic Stress Disorder
PMTE: Pre-migration traumatic experiences
UNHCR: United Nations High Commissioner for Refugees

## Footnotes

1 Female/Male, number of participant.

2 Age group, defined as following: [1]—20–25 yrs, [2]—25–30 yrs, [3]—30–35 yrs, [4]—35–40 yrs, [5]—40–45 yrs, [6]—45–50 yrs.
