# Supplementary material for: “Of course, you get depression in this situation”: Explanatory Models (EMs) among Afghan refugees in camps in Northern Greece
Source: BMC Psychiatry. 2023 Feb 27;23:125. doi: 10.1186/s12888-023-04613-2 (PMC9968643; doi:10.1186/s12888-023-04613-2)
Supplement: Supplementary file 1 — Additional file 1. [file 12888_2023_4613_MOESM1_ESM.docx]

**Supplementary Material**

| **Table S1. Vignette (F/M, Afghan)** |
| --- |
| *Aadela/Adel is 45 years old and* ***single parent from Afghanistan****,* ***living with 3 children.*** *One of the children has also been showing irritability and withdrawal, not wanting to speak to any friends and being regularly irritated with Aadela/Adel. Aadela/Adel stays in a camp in Greece, about 30- minute walk from the closest village. Aadela/Adel lost her/his husband/wife in Afghanistan 2 years ago during an armed conflict. So far, they are at the camp for 1 year and their interview for the asylum will take place in 10 months. Aadela/Adel is really worried about the outcome of the interview, and she/he is afraid that her/his application might be rejected. For the past 3 months, Aadela/Adel has been sleeping more than usual, having a difficult time to get out of bed and take care of herself/himself as well as her/his children like she/he used to. Her/his children also attend informal education classes at the camp. The teachers are concerned, and they fear that Aadela/Adel might be neglecting them. Sometimes her/his children are not prepared or on time for classes and many times she/he forgets to pick them up on time. Coming to Greece, Aadela/Adel has been really looking forward to get her/his children to school, as well as go on studying Greek herself/himself since where she/he grew up, she/he did not have the chance to finish school. Aadela/Adel also used to enjoy playing with her/his children, but for the last month she does not want to play with them anymore. Aadela/Adel also has a sibling that stays in the same camp who is really concerned about her/him and sometimes he/she comes to help her/him clean her/his place. Aadela/Adel said to her brother/his sister that she/he feels really tired all the time and many times she/he cries when she/he is by herself/himself feeling very sad and not being able to “stop thinking”. Aadela/Adel feels that it is her/his fault that she/he cannot enjoy playing with her/his children because she/he is a “bad mother/father”.* |
|  |

| **Table S2. Interview Guide** |
| --- |
| The main questions were: *“What, if anything, do you think is wrong with Aadela/Adel?, What could be the reason for why Adela/Adel is feeling the way s/he does?, If you were her/his friend, what would you recommend her/him to do? And why? If you were in the country of origin, would you suggest him/her to do anything differently? How? How do you think the situation will improve for Aadela/Adel? If not, what do you think will prevent her from getting better?”.* Ultimately, a question was added with a resilience-based approach. “*What can give confidence and hope to Aadela/Adel about the future?”*. |

**Table S3. Characteristics of the focus-group meetings**

| Focus-group | Gender of the participants | Date | Settings | Mode of communication |
| --- | --- | --- | --- | --- |
| 1 | Male | July 2021 | Camp | Hybrid (ZOOM software) |
| 2 | Female | July 2021 | Camp | Online (Whatsapp Software) |
| 3 | Female | July 2021 | Camp | Online (Whatsapp Software) |
| 4 | Male | August 2021 | Community | Hybrid (ZOOM software) |
| 5 | Female | October 2021 | Camp | Physical meeting |
| 6 | Male | November 2021 | Camp | Physical meeting |
